# Association of TGF-β1 Polymorphism and TGF-β1 Levels With Chronic Hepatitis C and Cirrhosis: A Systematic Review and Meta-Analysis

**DOI:** 10.7759/cureus.41157

**Published:** 2023-06-29

**Authors:** VPS Punia, Nikhil Agrawal, Akash Bharti, Shaavi Mittal, Dhirender Chaudhary, Atmika Mathur, Shahzad Anwar, Aditya Chakravorty

**Affiliations:** 1 Internal Medicine, School of Medical Sciences and Research (SMSR) Sharda University, Greater Noida, IND; 2 Internal Medicine, University College of Medical Sciences, Delhi, IND; 3 Internal Medicine, FH Medical College, Agra, IND; 4 Internal Medicine, Bharati Vidhyapeeth Medical College, Pune, IND

**Keywords:** tgf-β1 polymorphism, hepatic fibrosis, hepatitis c, cirrhosis, liver

## Abstract

Despite the extensive research conducted on the relationship between transforming growth factor-beta 1 (TGF-β1) polymorphisms and levels and the onset and development of liver disease, there are still certain gaps that need to be addressed. To address these gaps and provide a comprehensive overview of the current knowledge, this review aimed to identify relevant published research on TGF-β1/TGF-β1 polymorphism, TGF-β1/TGF-β1 levels, and their associations with cirrhosis and hepatitis C. The synthesis of available data was performed to further enhance our understanding in this area. Adopting the Preferred Reporting Items for Systematic Reviews and Meta-Analysis (PRISMA) guidelines, a search strategy was implemented across several online databases to search for relevant articles as per the defined selection criterion. Eight studies were selected after the completion of the search strategy. Of the eight studies, five revealed a considerably high level of TGF-β1 in patients who had hepatitis C virus (HCV) and liver cirrhosis caused by hepatocellular carcinoma (HCC). The forest plot analysis showed a statistically significant impact of TGF-β1 polymorphism and levels on the incidence of hepatic cirrhosis and hepatitis C, with an odds ratio (OR) of 0.65 and a risk ratio (RR) of 0.76. The heterogeneity test showed a high level of heterogeneity at 94% and 95% for OR and RR, respectively, but the overall effect was significant with *P *< 0.01 for both measures. According to the results obtained, the authors concluded that TGF-β1 polymorphism and its associated levels should be taken into account while developing preventive and therapeutic approaches for hepatic cirrhosis and hepatitis C.

## Introduction and background

The liver, one of the most remarkable organs of the human body, serves as the repository for vital micronutrients and plays a critical role in erythropoiesis and the digestive process [1]. It also serves as the primary detoxifier in our body, acting in conjunction with the kidneys to purify and remove toxic metabolites generated from different metabolic pathways [2]. It has been further documented that hepatocytes possess the ability to regenerate and can take a lot of damage throughout an individual’s lifetime [3]. However, several systematic diseases can impair liver function and, in critical cases, may warrant the need for a liver transplant, the absence of which might prove to be fatal [3]. Cirrhosis and hepatitis C happen to be two such conditions that pose a serious threat to liver health [4]. An estimated 2 million people in the United States alone succumb to liver disease, with a million of these patients succumbing to complications related to cirrhosis and hepatitis C, respectively [5].

Liver cirrhosis is a condition where the liver becomes scarred and hardened due to excessive fibrosis, leading to poor liver function and unregulated hepatocellular apoptosis [4]. The primary causes of cirrhosis include excessive alcohol consumption and chronic viral hepatitis, particularly hepatitis B and C [6]. Other causes of cirrhosis include obesity, non-alcoholic fatty liver disease (NAFLD), and certain autoimmune disorders [6]. In the United States, it is estimated that approximately 2.4 million people are living with chronic hepatitis C, and it is responsible for more deaths than HIV [7]. Hepatitis C primarily spreads through contact with infected blood, most commonly through sharing needles or other drug injection equipment [8]. It can also be spread through sexual contact with an infected person or from mother to baby during childbirth [8].

Genetic influences have been implicated to play a significant role in the development of liver disease [9-10]. One such example of a genetic influence on liver disease is the hereditary hemochromatosis gene, which causes the body to absorb too much iron from food [11]. Over time, this excess iron can accumulate in the liver and other organs, leading to liver damage and an increased risk of liver cancer. Another example is alpha-1 antitrypsin deficiency, which is a genetic condition that affects the production of a protein called alpha-1 antitrypsin [12]. This protein plays a role in protecting the liver and lungs from damage caused by inflammation. In individuals with alpha-1 antitrypsin deficiency, the protein is not produced in sufficient quantities, leading to an increased risk of liver disease and lung disease [13].

The transforming growth factor-beta 1 (TGF-β1) is a cytokine that plays an important role in the pathogenesis of liver disease [9-10]. Several studies have investigated the association between genetic variations in TGF-β1 and the levels of TGF-β1 with the development and progression of liver disease [9-10,14-15]. TGF-β1 polymorphisms are associated with an increased risk of liver fibrosis and cirrhosis in patients with chronic hepatitis C virus (HCV) infection [14-15]. Specifically, the TGF-β1-509 C/T polymorphism is associated with increased TGF-β1 levels and increased risk of liver fibrosis and cirrhosis in HCV-infected patients [14-15].

However, while several studies have investigated the association between TGF-β1 polymorphisms and TGF-β1 levels with the development and progression of liver disease, there are still some literature gaps that need to be addressed. For instance, there is a need for more large-scale studies with diverse populations to further elucidate the association between TGF-β1 polymorphisms and liver disease. Additionally, more studies are needed to examine the impact of TGF-β1 levels on the progression of liver disease, including cirrhosis and hepatitis C. Therefore, more research is needed to elucidate the precise mechanisms by which TGF-β1 contributes to liver disease progression. Hence, using this systematic review and the subsequent meta-analysis, we aimed to identify relevant studies published on TGF-β1 polymorphism and TGF-β1 levels and their association with cirrhosis and hepatitis C and synthesize the evidence and provided an overview of the current state of knowledge.

## Review

Materials and Methods

**Figure 1:**
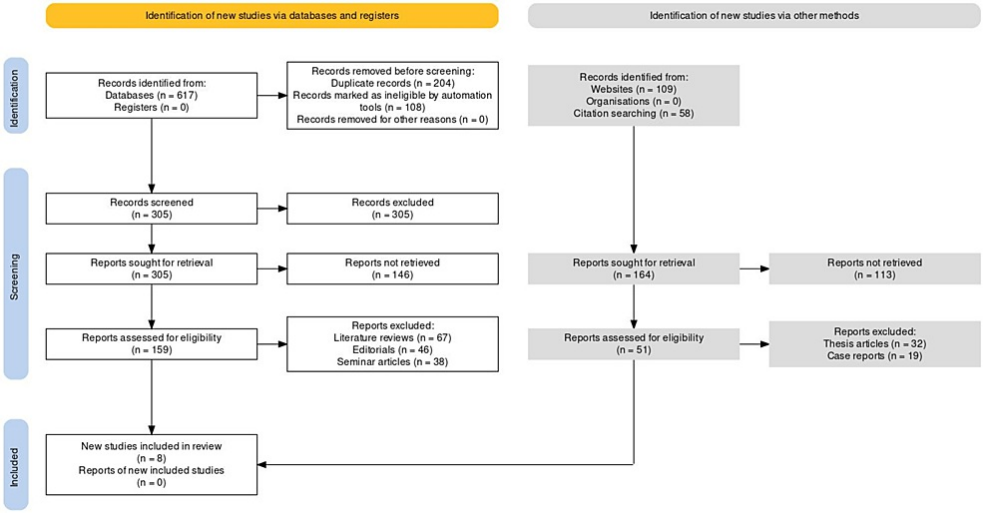
Flowchart displaying the study selection steps for this review. PRISMA, Preferred Reporting Items for Systematic Reviews and Meta-Analysis

The Preferred Reporting Items for Systematic Reviews and Meta-Analyses (PRISMA) protocol was a valuable tool for this systematic review [16-17]. This protocol is a widely used guideline for the reporting of systematic reviews and meta-analyses, and it provides a structured and transparent approach to conducting and reporting these types of studies. To adhere to the PRISMA protocol, the reviewers started by conducting a thorough search of the literature to identify all relevant studies on TGF-β1 polymorphism and TGF-β1 levels and their association with cirrhosis and hepatitis C, as represented in Figure 1. The search strategy was documented and reported according to the PRISMA guidelines.

PICOS Strategy

The PICOS strategy was used to develop the research question and inclusion criteria for this study. The population (P) of interest was patients with cirrhosis or hepatitis C. The intervention (I) was the presence of TGF-β1 polymorphism and the exposure (E) was TGF-β1 levels. The comparison (C) was the absence of TGF-β1 polymorphism or lower TGF-β1 levels. The outcomes (O) of interest were the association between TGF-β1 polymorphism and TGF-β1 levels with cirrhosis and hepatitis C progression, as well as the incidence and severity of complications associated with these conditions.

Search Protocol

To conduct a comprehensive search for relevant studies on the association between TGF-β1 polymorphism and TGF-β1 levels on cirrhosis and hepatitis C, a search strategy was developed using MeSH keywords across five databases, including PubMed, Embase, Cochrane Library, Scopus, and Web of Science. The search used a combination of MeSH terms such as "Transforming Growth Factor beta 1," "Polymorphism, Genetic," "Cirrhosis," "Hepatitis C," and "Liver Diseases." Keywords such as "TGF-beta 1," "polymorphism," "cirrhosis," "hepatitis C," "liver disease," and "genetic variations" were also included. The search strategy used Boolean operators such as "AND" and "OR" to combine the MeSH terms and keywords to obtain a relevant set of studies. The search was limited to studies conducted in humans and published in the English language from 2005 to the present. A manual search of the reference lists of the included articles was also conducted to identify additional relevant studies. The search strategy was designed to ensure that all relevant studies were captured and minimize the risk of missing any important studies (Table 1).

**Table 1:** Tabular representation using MeSH keywords.

Database	Search Terms
PubMed	(("Transforming Growth Factor beta1"[Mesh]) OR ("TGF-beta1"[Mesh]) OR ("TGF-beta1"[Title/Abstract])) AND (("Polymorphism, Genetic"[Mesh]) OR ("Polymorphism"[Mesh]) OR ("Genetic Variation"[Mesh]) OR ("SNP"[Title/Abstract])) AND (("Hepatitis C"[Mesh]) OR ("HCV"[Title/Abstract])) AND (("Cirrhosis"[Mesh]) OR ("Liver Cirrhosis"[Mesh]) OR ("Liver Fibrosis"[Mesh]) OR ("Hepatic Fibrosis"[Mesh]) OR ("Hepatic Cirrhosis"[Mesh]))
Embase	('transforming growth factor beta1'/exp OR 'tfg-beta1'/exp OR 'tfg-beta1') AND ('polymorphism'/exp OR 'polymorphism' OR 'genetic variation'/exp OR 'genetic variation' OR 'snp') AND ('hepatitis c'/exp OR 'hcv') AND ('cirrhosis'/exp OR 'liver cirrhosis'/exp OR 'liver fibrosis'/exp OR 'hepatic fibrosis'/exp OR 'hepatic cirrhosis'/exp)
Scopus	(TITLE-ABS-KEY("Transforming Growth Factor beta1") OR TITLE-ABS-KEY("TGF-beta1")) AND (TITLE-ABS-KEY("Polymorphism") OR TITLE-ABS-KEY("SNP") OR TITLE-ABS-KEY("Genetic Variation")) AND (TITLE-ABS-KEY("Hepatitis C") OR TITLE-ABS-KEY("HCV")) AND (TITLE-ABS-KEY("Cirrhosis") OR TITLE-ABS-KEY("Liver Cirrhosis") OR TITLE-ABS-KEY("Liver Fibrosis") OR TITLE-ABS-KEY("Hepatic Fibrosis") OR TITLE-ABS-KEY("Hepatic Cirrhosis"))
Web of Science	TS=("Transforming Growth Factor beta1" OR "TGF-beta1") AND TS=("Polymorphism" OR "SNP" OR "Genetic Variation") AND TS=("Hepatitis C" OR "HCV") AND TS=("Cirrhosis" OR "Liver Cirrhosis" OR "Liver Fibrosis" OR "Hepatic Fibrosis" OR "Hepatic Cirrhosis")
Cochrane Library	(((("Transforming Growth Factor beta1"[MeSH Terms]) OR ("TGF-beta1"[MeSH Terms]) OR ("TGF-beta1"[Title/Abstract]))) AND ((("Polymorphism, Genetic"[MeSH Terms]) OR ("Polymorphism"[MeSH Terms]) OR ("Genetic Variation"[MeSH Terms]) OR ("SNP"[Title/Abstract])))) AND ((("Hepatitis C"[MeSH Terms]) OR ("HCV"[Title/Abstract]))) AND ((("Cirrhosis"[MeSH Terms]) OR ("Liver Cirrhosis"[MeSH Terms]) OR ("Liver Fibrosis"[MeSH Terms]) OR ("Hepatic Fibrosis"[MeSH Terms]) OR ("Hepatic Cirrhosis"[MeSH Terms]))))

Inclusion and Exclusion Criteria

The inclusion and exclusion criteria were established to ensure that all studies included in the systematic review were relevant to the research question and met the eligibility criteria. The inclusion criteria were studies that investigated the association between TGF-β1 polymorphism and TGF-β1 levels on cirrhosis and hepatitis C-like liver diseases. The studies had to be published in the English language and involved human subjects. The exclusion criteria were studies that did not meet the inclusion criteria, studies that were not conducted on human subjects, studies with irrelevant outcomes, animal studies, review articles, conference abstracts, case reports, and studies with insufficient data. Studies that were duplicates were also excluded. The screening of studies for inclusion and exclusion was carried out in two stages. The first stage involved screening the titles and abstracts of all studies identified in the search. In the second stage, full-text articles were assessed to determine their eligibility for inclusion.

Reviewer Strategy

For this study, a team of two reviewers was involved in the screening of articles. Both reviewers were trained in the inclusion and exclusion criteria and were familiar with the PICOS strategy. Initially, the reviewers independently screened the titles and abstracts of all articles identified through the search strategy to determine whether they met the inclusion criteria. The reviewers then met to discuss the results and resolve any discrepancies. Next, the full texts of potentially relevant articles were retrieved and independently assessed by the reviewers. Any disagreements were resolved by consensus or by consulting a third reviewer. Finally, the reviewers evaluated the quality of the included studies using the appropriate critical appraisal tool and extracted the necessary data for the analysis.

Bias Assessment

**Figure 2:**
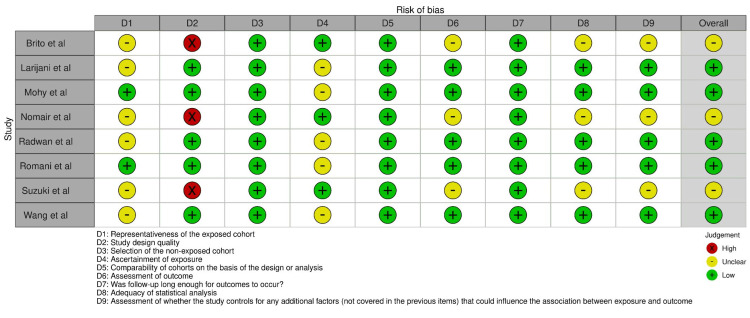
Bias evaluation across different domains for the included studies. Studies by de Brito et al. [20], Larijani et al. [21], Mohy and Fouad [22], Nomair et al. [23], Radwan et al. [24], Romani et al. [25], Suzuki et al. [26], Wang et al. [27].

For this investigation, the Newcastle-Ottawa Scale (NOS) tool was used for bias assessment [18-19]. Two reviewers independently assessed the quality of the selected studies. In the selection domain, the studies were evaluated based on the representativeness of the exposed cohort, selection of the non-exposed cohort, ascertainment of exposure, and demonstration that the outcome of interest was not present at the start of the study (Figure 2). The comparability domain assessed the comparability of the cohorts based on factors such as age, gender, and other potential confounding variables. Finally, the outcome domain evaluated the assessment of the outcome of interest, the adequacy of follow-up, and the assessment of the follow-up.

*Meta-Analysis Protocol* The meta-analysis protocol for this study was established using the Review Manager (RevMan) 5 (The Nordic Cochrane Center, Odense, Denmark) software. The forest plots for the odds ratio (OR) and the risk ratio (RR) were generated using a fixed-effects model with 95% confidence intervals (CI). The studies included in the meta-analysis were evaluated for homogeneity and statistical significance using the chi-square test and the *I*^2^ statistics, respectively. The level of statistical significance was set at *P *< 0.05. The overall effect size was calculated using the Mantel-Haenszel method. The meta-analysis aimed to evaluate the association between TGF-β1 polymorphism and TGF-β1 levels in patients with cirrhosis and hepatitis C and determine the overall effect size of this association. The protocol ensured that the meta-analysis was conducted using a standardized and transparent approach, which is important for maintaining the quality and reliability of the findings.

Results

Table 2 presents the characteristics of all eight studies that were deemed to be relevant for inclusion in this systematic review and meta-analysis. The table consists of eight studies with information on the year, region, sample size, groups assessed, gender ratio, mean age, and hepatitis assessment tool used in each study. The studies were conducted in Brazil, Iran, Egypt, and Japan between 2003 and 2021. The sample sizes ranged from 70 to 440 participants, and the assessment tool used to measure hepatitis was enzyme-linked immunosorbent assay (ELISA) in all the studies. The study groups assessed included HCV patients, healthy controls, cirrhotic groups, non-cirrhotic groups, and patients with hepatocellular carcinoma (HCC). The gender ratio varied across studies, with the majority of participants being male. The mean age of participants ranged from 38.47 to 59.5 years. These characteristics were used in the systematic review and meta-analysis to compare the findings across the studies and assess their overall effect on TGF-β1 polymorphism and TGF-β1 levels in cirrhosis and hepatitis C.

**Table 2:** Characteristics of the included studies in terms of their demographics. HCV, hepatitis C virus; HCC, hepatocellular carcinoma; ELISA, enzyme-linked immunosorbent assay

Study ID	Year	Region	Sample size (*n*)	Groups assessed	Gender ratio	Mean age (years)	Hepatitis assessment tool
de Brito et al. [20]	2020	Brazil	399	99 HCV patients and 300 healthy controls	52 males in the HCV group	>18 (range)	ELISA
Larijani et al. [21]	2016	Iran	165	89 patients with HCV and 76 healthy controls	112 males	38.47	ELISA
Mohy and Fouad [22]	2014	Egypt	80	40 in cirrhotic group and 40 in non-cirrhotic group	50 males	40-60 (range)	ELISA
Nomair et al. [23]	2021	Egypt	70	34 in HCC and 36 in non-HCC group	40 males	59.5 ± 6.9	ELISA
Radwan et al. [24]	2012	Egypt	440	152 HCV and cirrhotic patients, 128 HCV and HCC patients, and 160 healthy controls	236 males	58 ± 12.15	ELISA
Romani et al. [25]	2011	Iran	333	164 in cirrhotic group and 169 in non-cirrhotic group	217 males	44.4 ± 15.65	ELISA
Suzuki et al. [26]	2003	Japan	307	206 patients with HCV and 101 healthy controls	170 males	60 ± 12	ELISA
Wang et al. [27]	2005	China	260	210 patients with HCV and 50 healthy controls	Unspecified	Unspecified	ELISA

Table 3, on the other hand, provides a summary of eight selected studies that have examined the association between certain biomarkers and genes with HCV infection, cirrhosis, and/or HCC. Each study included in the table employed a case-control or cross-sectional design, and polymerase chain reaction-restriction fragment length polymorphism (PCR-RFLP) was used as a method to assess polymorphisms. The biomarkers that were assessed in the studies include ALT, AST, GGT, AFP, PT, TG, INR, CBC, and total bilirubin count, while the genes that were assessed include TGF-β1, interferon-gamma (IFN-γ), Patatin-like phospholipase domain-containing protein 3 (PNPLA3), and tumor necrosis factor-alpha (TNF-α). The polymorphisms assessed include -509 C/T, +874A/T, rs1800469 (C/T), Arg25Pro, and +869 C/T, among others. The results of the studies are varied. Brito et al found a higher risk of infection and the frequency of the TGFB1 -509C/T polymorphism genotype was linked to HCV infection [20]. Larijani et al. found no significant correlation between the TGF-β1-509 polymorphism and susceptibility to HCV infection based on allele frequency, but there was a significant difference between the C and T alleles of HCV patients [21]. Mohy and Fouad found that the 509 CC and TT alleles were significantly more expressed in cirrhotic patients than non-cirrhotic group [22]. Nomair et al. found that both TGF-β1 levels and GG allele frequency were significantly higher in the cirrhosis patients than in the control group [23]. Radwan et al. found that the frequencies of TGF-β1-509 TT, TNF-α-308 AA, and GA alleles were significantly higher in cirrhotic and HCC groups [24]. Suzuki et al. [26] found that the TGF-β1 polymorphism at codon 10 and the hepatic fibrosis levels were not significantly correlated, while Wang et al. [27] found that TGF-β1 allelic variants were more common in Caucasians evaluated, and the presence of prolines in codon 25 or 10 was linked to the interindividual variability in the development of more severe fibrosis in the HCV group.

**Table 3:** Selected studies and the assessment pertaining to the impact of polymorphisms observed in them. HCV, Hepatitis C virus; HCC, hepatocellular carcinoma; IFN-γ, interferon-gamma; TNF-α, tumor necrosis factor-alpha; PCR-RFLP, polymerase chain reaction-restriction fragment length polymorphism; AFP, alpha-fetoprotein; AST, aspartate aminotransferase; ALT, alanine aminotransferase; PT, prothrombin time; TG, triglycerides; INR, international normalized ratio; CBC, complete blood count

Study ID	Protocol	Biomarkers assessed	Genes assessed	Polymorphism assessment method	Polymorphisms assessed	Inference assessed
de Brito et al. [20]	Cross-sectional	ALT, AST, and GGT	TGF-β1 and IFN-γ	PCR-RFLP	-509 C/T and +874A/T	A higher risk of infection and the frequency of the TGF-β1 -509C/T polymorphism genotype were linked to HCV infection. In the control and HCV groups, plasma levels of IFN-γ were higher in TT genotype carriers. The HCV group and TT genotype carriers had the highest TGF-β1 levels. TGF-β1 levels were higher in those with cirrhosis.
Larijani et al. [21]	Case-control	AFP, AST, ALT, PT, TG, INR, CBC, and total bilirubin count	TGF-β1-509	PCR-RFLP	rs1800469 (C/T)	No significant correlation between polymorphism and susceptibility to HCV infection could be found based on allele frequency, but there was a significant difference between the C and T alleles of HCV patients.
Mohy and Fouad [22]	Case-control	AFP, AST, ALT, PT, TG, INR, CBC, and total bilirubin count	TGF-β1	PCR-RFLP	-509 C/T	The 509 CC and TT alleles were found to be significantly more expressed in cirrhotic patients (52.5% and 25%) than non-cirrhotic groups (10% and 7.5%)
Nomair et al. [23]	Case-control	AFP, AST, ALT, PT, TG, INR, CBC, and total bilirubin count	TGF-β1 (Arg25Pro) and PNPLA3 (I148M)	PCR-RFLP	GG and GC for both the genes	Both TGF-β1 levels and GG allele frequency were significantly higher in the cirrhosis patients than the control group
Radwan et al. [24]	Case-control	AFP, AST, ALT, PT, TG, INR, CBC, and total bilirubin count	TGF-β1-509 and TNF-α-308	PCR-RFLP	-509 C/T and 308G/G	In comparison to the healthy control groups, the frequencies of TGF-β1-509 TT, TNF-α-308 AA, and GA alleles were significantly higher in cirrhotic and HCC groups.
Romani et al. [25]	Case-control	Unspecified	TGF-β1 (and its other two isoforms TGF-β2 and TGF-β3)	PCR-RFLP	-509 C/T, +869 C/T, and +915 G/C	No significant differences were observed between the cirrhotic and non-cirrhotic group in terms of TGF-β1 levels and polymorphisms
Suzuki et al. [26]	Case-control	ALT	TGF-β1	PCR-RFLP	T/T, C/T, and C/C	The TGF-β1 polymorphism at codon 10 and the hepatic fibrosis levels were not significantly correlated. In contrast, neither healthy controls nor individuals with HCV had any genetic changes to codon 25.
Wang et al. [27]	Case-control	Unspecified	TGF-β1	PCR-RFLP	Codons 10, 25, and 263	TGF-β1 allelic variants were more common in the Caucasians evaluated, and the presence of prolines in codon 25 or 10 was linked to the interindividual variability in the development of more severe fibrosis in the HCV group.

**Figure 3:**
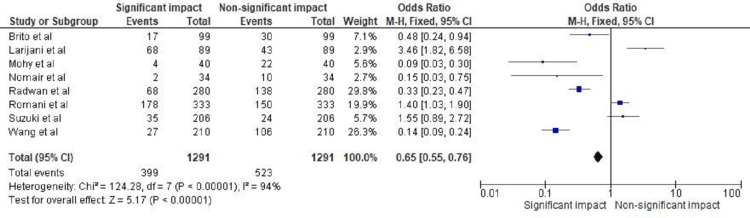
Impact of TGF-β1 polymorphism and levels on incidence of hepatic cirrhosis and hepatitis C in terms of OR. Studies by de Brito et al. [20], Larijani et al. [21], Mohy and Fouad [22], Nomair et al. [23], Radwan et al. [24], Romani et al. [25], Suzuki et al. [26], Wang et al. [27]. TGF-β1, transforming growth factor-beta 1; OR, odds ratio

The forest plot in Figure 3 presents the statistical analysis of the impact of TGF-β1 polymorphism and levels on the incidence of hepatic cirrhosis and hepatitis C. The OR was found to be 0.65 with a 95% CI ranging from 0.55 to 0.76, indicating a statistically significant impact of TGF-β1 polymorphism and levels on the incidence of the disease. The heterogeneity test showed a chi-square value of 124.28 with 7 degrees of freedom (df), and the *P*-value <0.01. Additionally, the *I*² statistic indicated a high level of heterogeneity at 94%. The test for overall effect showed a *Z*-value of 5.17, with a *P*-value <0.01, further supporting the significance of the observed impact. In conclusion, this analysis suggests that TGF-β1 polymorphism and levels have a significant impact on the incidence of hepatic cirrhosis and hepatitis C.

**Figure 4:**
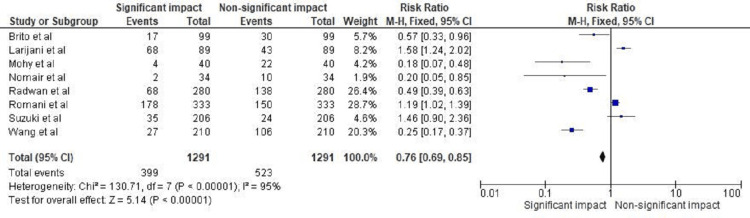
Impact of TGF-β1 polymorphism and levels on incidence of hepatic cirrhosis and hepatitis C in terms of RR Studies by de Brito et al. [20], Larijani et al. [21], Mohy and Fouad [22], Nomair et al. [23], Radwan et al. [24], Romani et al. [25], Suzuki et al. [26], Wang et al. [27]. TGF-β1, transforming growth factor-beta 1; RR, risk ratio

The statistical analysis for the forest plot in Figure 4 shows an RR of 0.76 (0.69, 0.85) for the significant versus nonsignificant impact of TGF-β1 polymorphism and levels on the incidence of hepatic cirrhosis and hepatitis C. The heterogeneity of this analysis was chi² = 130.71, with df = 7 and *P* < 0.01, and *I*² = 95%. The test for overall effect was *Z* = 5.14, with *P* < 0.01. These results indicate a significant impact of TGF-β1 polymorphism and levels on the incidence of hepatic cirrhosis and hepatitis C. The analysis also suggests that there is a high degree of heterogeneity in the results, indicating potential differences between the studies included in the analysis. Despite this, the overall effect was significant, with a relatively small confidence interval, suggesting that the impact of TGF-β1 on the incidence of hepatic cirrhosis and hepatitis C is consistent across the studies included in the analysis.

Discussion

This study aimed to explore the association between certain biomarkers and genes with HCV infection, cirrhosis, and/or HCC. The results of this study are significant in that they demonstrate a statistically significant impact of TGF-β1 polymorphism and levels on the incidence of hepatic cirrhosis and hepatitis C. The forest plots in Figures 3-4 present the statistical analysis of the impact of TGF-β1 polymorphism and levels on the incidence of the disease. The analysis showed a significant impact of TGF-β1 polymorphism and levels on the incidence of the disease, with a relatively small CI, suggesting that the impact of TGF-β1 on the incidence of hepatic cirrhosis and hepatitis C is consistent across the studies included in the analysis. This study has addressed some of the literature gaps in this respect, as it provides a summary of eight selected studies that have examined the association between certain biomarkers and genes with HCV infection, cirrhosis, and/or HCC. The results of the studies are varied, highlighting the need for further research in this area. The findings of this study have important implications for future research in this field. The results suggest that TGF-β1 polymorphism and levels play a significant role in the incidence of hepatic cirrhosis and hepatitis C. Further research could focus on investigating the mechanisms by which TGF-β1 polymorphism and levels contribute to the development of these conditions, as well as exploring potential therapeutic strategies that target TGF-β1. In addition, future research could focus on exploring the potential impact of other biomarkers and genes on the development of hepatic cirrhosis and hepatitis C. Overall, this study provides important insights into the role of TGF-β1 in the development of hepatic cirrhosis and hepatitis C and highlights the need for further research in this area.

Several studies that examined the TGFB1 -509C/T polymorphism in HCV infection did not quantify viral load, although variations in viral load have been linked to another polymorphism in the TGF-β1 gene [14-30]. As seen in this review, TGF-β1 was considerably higher in patients with HCV infection and in people with the TT genotype from both the control and HCV groups. According to one study [31], the TGF-β1-509C/T polymorphism is linked to greater levels of TGF-β1. A couple of studies [32-33] found a link between high levels of TGF-β1 and the loss of hepatocytes, a sign of disease development. According to immunohistochemistry, the prevalence of liver injury is closely correlated with the number of TGF-β1-positive cells. According to a rodent-based study [34], altered TGF-β1 was linked to latent TGF-β1 binding proteins that can lead to tumors and inflammation. A crucial factor in defining the switch in the TGF-β1 signaling pathway from tumor suppression to fibrogenesis, which accelerates liver fibrosis and raises the risk for HCC, is also the persistence of chronic inflammation, as seen in chronic viral hepatitis. As a result, TGF-β1 variations will change the degree of protein expression, which may have an impact on a person's susceptibility to developing tumors, including HCC as observed in one of the studies that we selected for our review [24]. Several articles have observed that the TGF-β1 signaling system exhibits strong pro-oncogenic features, and disruption of the route is frequently seen in a variety of chronic liver disorders, fibrogenesis, and liver cancer formation. TGF-β1 signaling changes were also linked to unfavorable tumor biology [35-37].

This study has several limitations. First, the studies included display varied results, making it difficult to draw a definitive conclusion regarding the impact of the biomarkers and genes assessed on HCV infection, cirrhosis, and HCC. Additionally, the studies included in the table only employ case-control or cross-sectional designs, which may limit the generalizability of the findings. Furthermore, the meta-analysis shows a high level of heterogeneity between the studies included in the analysis. This may be due to differences in sample sizes, methodologies, and populations studied. Therefore, the results of the analysis may not be generalizable to other populations or settings. Hence, while the study provides valuable insights into the potential impact of TGF-β1 polymorphism and levels on the incidence of hepatic cirrhosis and hepatitis C, its limitations should be considered when interpreting the results.

## Conclusions

Conclusively speaking, this study analyzed the impact of TGF-β1 polymorphism and levels on the incidence of hepatic cirrhosis and hepatitis C. The findings of the meta-analysis showed a statistically significant impact of TGF-β1 polymorphism and levels on the incidence of the disease. These findings suggest that TGF-β1 polymorphism and levels are important factors to consider in the development of preventive and treatment strategies for hepatic cirrhosis and hepatitis C. However, this review warrants the fact that further studies are required to understand the potential therapeutic targets for these diseases due to the mixed assessments observed.
